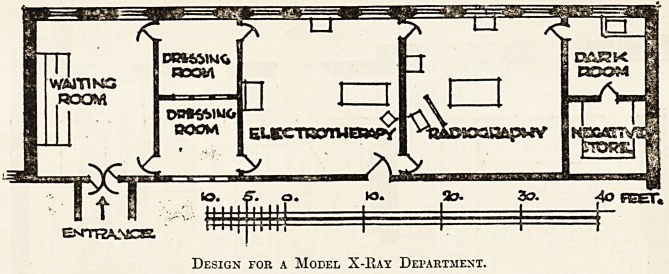# Electro-Therapy and Radiography

**Published:** 1911-06-03

**Authors:** 


### Hospital Architecture and Construction.

[Communications on this subject should be marked "Architecture" in the left-hand top corner of the envelope.]

### ELECTRO-THERAPY AND RADIOGRAPHY.

The science of curing skin diseases by electric processes and the application of $x$-rays for surgical purposes have made vast strides in recent years, yet in many establishments the rooms used for such purposes have been selected rather because they were the rooms most easily spared than for any suitability in plan or design. The time has certainly come when these important departments should receive special architectural attention in all modern hospitals.

### A Proposed Plan.

The following plan indicates a convenient disposition of the necessary accommodation. There should be a subordinate waiting-room contiguous to the general out-patients' waiting-hall where the patients may rest till the operator is ready for them. It will be seen that this room is shown about 20 feet by 15 feet, and it is desirable that it should lead on to male and female dressing-rooms. These respective dressing-rooms in turn lead on to the electro-therapy chamber. This should be about 20 feet by 20 feet, and should be well lighted, but may have mechanical darkening blinds. It accommodates the Finsen or Kromayer light appliances, a Pantostat, and possibly a pulsating sinusoidal machine. If the last-named is included in the installation a hip bath might with advantage be provided so that the patient may be immersed for treatment of the trunk muscles. For limb treatment probably two jars would be sufficient, but as these would require filling and emptying at a sink it will be simpler to provide a hip bath connected to drains in the usual way.

It is also very desirable to have a porcelain sink about 30 inches by 18 inches, with the taps not less than 12 or 14 inches above it; this is for the general convenience of operators and nurses.

### The Radiographic Room.

A glance at the plan will show that doors lead from the electro-therapy room into the radiography chamber, which will be large enough for one installation if made 20 feet square, and it may here be said that it is desirable only to have one installation to one room, and further, that the enclosing wall should be of not less than 9-inch thickness, with the doors placed in the corners of the room so as to prevent rays from penetrating the woodwork of the doors instead of being caught by the walls. A sink will also prove useful in this room. The room need not necessarily be well lighted, but it must have blinds which effectively darken the room and can be easily manipulated by the operator.

### General Considerations.

The following remarks apply equally to both the electro-therapy and radiography rooms. It is desirable to provide earth connections where electricity is so much used, and it is also necessary to have the floors well insulated. Both these rooms should have as much cupboard space as possible; a cupboard 18 inches high with its underside some 6 feet above the floor will not be out of place, and its top will form a useful shelf for various instruments and globes. It is almost impossible to have too many wall plugs for electric connections in these rooms, as the various appliances have to be used in so many different positions.

Design for a Model X-Ray Department.

The greatest care should be taken by the designer of these departments to maintain them at an equable temperature and to provide adequate means of ventilation; nothing is of more consequence, and it must be remembered that the patient may be almost entirely unclothed while at the same time the operator and nurses have to stay in the same atmosphere for probably several hours of the day.

It is likely that these departments will be under the charge of one honorary officer, and in planning the rooms it will be well to bear this in mind.

### Details.

Twelve feet would be ample height for these rooms, and all angles at floors, walls, and ceilings should be rounded to a 3-inch radius; the detail also of all doors and window finishings kept rounded and flat, free from mouldings or other ornamentation which might tend to accumulate dust. Probably a good cork linoleum would prove the most efficient floor-covering, comfortable to the occupants while at the same time proving an effective insulator. The radiators for heating should stand on cement bases about 3 inches high, rounded off in a manner similar to the floor skirting.

A further glance at the plan will show that a dark room is provided in connection with the radiograph room. Ten feet square will be sufficient for its size, and it should have at least one good sink deep enough for washing whole-plate negatives, and with drying racks over. It should be well lighted and have in its window a carefully fitted ruby blind (working in grooves) for developing purposes. Plenty of shelving will be useful again in this room, and the store for the careful keeping of negatives, probably indexed, will have shelving about 10 inches wide round three of its sides.

## Figures and Tables

**Figure f1:**